# Using silica nanoparticles to deliver antibiotics for treating Gram-positive bacterial infections in a 3D-bioprinted dermal model

**DOI:** 10.3389/fbioe.2026.1737616

**Published:** 2026-02-17

**Authors:** Thiago Antonio Moretti de Andrade, Ariyan Suleman, Kali Scheck, Ruchi Sharma, Claire Benwood, Alexandre Brolo, Stephanie M. Willerth

**Affiliations:** 1 Department of Mechanical Engineering, University of Victoria, Victoria, BC, Canada; 2 School of Medical Sciences, University of Victoria, Victoria, BC, Canada; 3 Department of Biomedical Engineering, University of Victoria, Victoria, BC, Canada; 4 Department of Chemistry, University of Victoria, Victoria, BC, Canada; 5 Axolotl Biosciences, Victoria, BC, Canada; 6 Centre for Advanced Materials and Technology, University of Victoria, Victoria, BC, Canada; 7 School of Biomedical Engineering, University of British Columbia, Vancouver, BC, Canada

**Keywords:** 3D bioprinting, antibiotics, drug delivery, fibroblasts, Gram-positive bacteria, nanomedicine, silica nanoparticle, skin human model

## Abstract

**Introduction:**

Bacterial antibiotic resistance has emerged as a significant global threat, making it increasingly challenging to effectively treat infections in patients. Nanomedicine technologies can be implemented for the targeted delivery of medications and drugs to patients. This work investigates the use of silica nanoparticles (SiNPs) loaded with the clindamycin and tetracycline antibiotics to treat Staphylococcus epidermidis infection in a 3D-bioprinted dermal model. SiNPs are stable, biocompatible, and can be loaded with small molecules like antibiotics.

**Methods:**

The SiNPs were synthesized and loaded with antibiotics. The loading efficiency of the SiNPs was determined by UV-Vis spectroscopy and high-performance liquid chromatography. Dome-shaped constructs containing fibroblasts were 3D printed using a fibrin-based bioink to mimic the dermis of skin. These constructs were then inoculated with bacterial cultures labeled with green fluorescent protein (GFP) 4 days post printing and then treated with antibiotic-loaded SiNPs to determine their effect on bacterial growth. After the incubation phase, the bacteria were cultured in broth to determine the colony-forming unit (CFU) count on toxin superantigen (TSA) plates containing 10 mg/mL chloramphenicol.

**Results and Discussion:**

The CFU count of the 3D-bioprinted human constructs samples treated with antibiotics was significantly lower than both the SiNP-treated and untreated samples. The results suggest that antibioticreleasing SiNPs can serve as a more efficient treatment for skin bacterial infections.

## 1 Introduction

Antibiotics are commonly used to control bacteria that cause severe illnesses and infections. Since the introduction of antibiotics, their subsequent mass production has led to a massive increase in bacterial resistance in microorganisms. The rapid and global dissemination of antibiotic resistance has been compounded by the lack of surveillance tools for monitoring its spread. The spread of antibiotic-resistant bacteria poses a serious problem for healthcare providers, as most medical interventions require antibiotics to reduce the risk of infection (Hutchings et al., 2019). The increase in the number of antibiotic-resistant bacteria is a major burden to the healthcare system. The World Health Organization estimates that by 2050, the number of deaths associated with drug-resistant bacteria will reach 10 million per year (Fulaz et al., 2020).

As antibiotics have become more commonly used in agriculture, hospital settings, and the over-prescription in communities for non-bacterial infections, certain bacteria have developed tolerance or resistance to the treatments by adapting to the targets of the antibiotics. The rising emergence of strains of *Staphylococcus aureus* that are resistant to all beta-lactam antibiotics, grouped as MRSA (methicillin-resistant *Staphylococcus aureus*), has caused hospitals to change their approach in how they detect and treat bacterial infections. The use of various antibiotics has caused more difficulty in the treatment of resistant microbes due to the alternative doses, as well as the higher doses needed (David and Daum, 2010). Members of the genus *Staphylococcus*—a group of Gram-positive bacteria—appear in a coccal shape and usually form clusters. Several of them are unable to cause disease and live commensally on the skin as part of the skin microbiota. *Staphylococcus epidermidis* can cause serious complications in catheters and surgical implants by forming biofilms (Devlin et al., 2021; Liu et al., 2022; Rahaman et al., 2023), which are structurally confluent layers of bacteria that form a protective layer for the microorganism (Archer et al., 2011).

Tetracycline hydrochloride (HCl), a broad-spectrum antibiotic, can treat both Gram-positive and Gram-negative bacteria intravenously and orally. Globally, an 8.7% resistance has been observed in *S. aureus* to methicillin. The acquisition of resistance can be due to several factors, including mobile genetic elements that carry tetracycline-specific resistance mutations, chromosomal mutations, or ribosomal binding site mutations. The efflux and enzymatic inactivation of tetracycline are the main mechanisms of action affected, leading to resistance and further success of the bacteria (Grossman, 2016). In *S. aureus*, tetracycline is inhibited in multidrug resistance by utilizing the MATE-family efflux pump. Little is known about the resistance in Gram-positive species, but overexpression of MepA leads to reduced susceptibility to the tetracycline antibiotic class (Devlin et al., 2021; Liu et al., 2022; Rahaman et al., 2023). The increased resistance due to efflux inhibition makes it an ideal candidate for inclusion into silica nanoparticles (SiNPs) due to an increased concentration that would have a longer saturation time and overcome the efflux pump. Tetracycline was also selected due to the conferred resistance that is present in the genome of the acquired strain from the American Type Culture Collection (ATCC).

The antibiotic clindamycin HCl can treat *S. aureus* infections in various locations, including strep throat, bone, and joints. It has been useful in the specific treatment of MRSA, specifically in skin and soft tissue infection (Shoji et al., 2015). Clindamycin can increase the incidence of *Clostridium difficile* infection (Devlin et al., 2021; Liu et al., 2022; Rahaman et al., 2023), which is detrimental to patients due to the severe impact that it can have on the normal bacterial flora. Accordingly, the use of oral antibiotics is only recommended as a last resort in certain cases where the patient needs emergency treatment.

The use of silica nanoparticles (SiNPs) has become increasingly popular for drug delivery applications. Using SiNPs to deliver hydrophobic drugs results in improved drug absorption by reducing degradation, as they have a protective effect on their encapsulated cargo. Drug loading allows the drugs to penetrate acutely into the pore channels of the carrier, thus allowing more contact time with a higher concentration of the drug of interest (Wang et al., 2015). Localized drug delivery can considerably decrease the microorganism count within an infection site, reducing the amount of antibiotic that must be delivered for treatment (Imran et al., 2022). Accordingly, nanotechnologies present a potential therapeutic way to combat antibiotic-resistant bacteria that pose a medical threat to patients by reducing the dose and providing localized delivery of antibiotics compared to systemic administration (Love et al., 2012). The size of the nanoparticles enables them to pass through cell membranes, but they might not be able to enter the cell nucleus. Larger particles would be ideal for human patients, as they will be less likely to cross membranes or affect cellular processes. The use of more stable nanoparticles that are made of safer and well-understood materials is the best approach to reducing the risk of exposure that could be harmful to the patient and impede cellular functions (Sukhanova et al., 2018). SiNPs present lower toxicity than other particles made of metals. SiNPs are useful for the high surface area that allows the easier incorporation of drugs at a similar weight to the particle itself at a ratio of 1:1 (Gulin-Sarfraz et al., 2019). The use of SiNPs as a drug carrier has the added advantage of being able to target the tissue/organ of choice and deliver a higher dosage of drug to the region of interest. This property is specifically of interest due to the prevalence of systemic side effects that antibiotics can cause, also correlating to a higher dosage that is necessary to effectively kill the pathogen. Using SiNPs as a carrier for antibiotics reduces the need for greater dosage treatment and longer patient follow-through (Mebert et al., 2016). Nanoparticle size also influences toxicity (Maccuaig et al., 2022; Watermann and Brieger, 2017). The use of nanoparticles can greatly enhance the antibiotic effectiveness by avoiding microbial detection, deactivating the biofilm formation, and increasing the concentration around the target cells (Fulaz et al., 2020). SiNPs can increase the distribution of antibiotics within bacterial growth colonies that are adherent to a surface (de la Fuente-Núñez et al., 2013). Targeted delivery can also reduce side effects in patients while increasing the local dosage. This form of therapy can be vital to hospitalized critical-care patients in hospitals with severe infections involving *S. aureus* and other pathogenic bacteria (Hussain et al., 2018).

Three-dimensional (3D) bioprinting can fabricate anatomically and functionally relevant skin constructs by depositing viable cells and tailored bioinks with spatial precision. The Willerth laboratory at the University of Victoria (Victoria, BC, Canada) was the first academic group to use the microfluidic Aspect Biosystems RX1 (Lab-on-a-Printer™) platform for bioprinting human stem cell-derived neural tissues with high viability and reproducibility using a novel fibrin-based bioink formulation (Abelseth et al., 2019; Benwood et al., 2023; de la Vega et al., 2018; Sharma et al., 2020). They have also recently bioprinted skin models using the Bio X extrusion-based bioprinter produced by CELLINK. Andrade et al. (2024) bioprinted a novel skin co-culture model using human keratinocytes and fibroblasts to mimic the dermal compartment of the skin, thereby laying the foundation for a model of the epidermis–dermis interface. This approach leverages the spatial control of bioprinting and the biomimetic environment of a tailored bioink to recreate the relevant dermal architecture and cell population *in vitro*. It also reported the rheological properties of the bioink used in the present study. Diaz et al. (2025) have used these tissues as a way to model infection with *Staphylococcus aureus* and *Staphylococcus epidermidis* to mimic bacterial skin disease with the fibrin-based bioink commercialized by Axolotl Biosciences. Jorgensen et al. (2023) integrated six distinct human dermal cell types into a multilayered skin construct using extrusion bioprinting. *In vitro* assessments revealed that this construct preserved a tri-layered architecture and underwent appropriate maturation. In murine models, the construct expedited the process of wound healing while facilitating neovascularization and the remodeling of the extracellular matrix, thereby mitigating fibrosis and scar formation. In porcine models, the application of an autologous porcine bioprinted skin construct resulted in enhanced epithelialization and diminished wound contraction, indicating that bioprinted skin may serve as a viable alternative for the treatment of human full-thickness wounds.

In this study, we used a bioprinted dermal tissue model consisting of fibroblasts as our platform for modeling bacterial infection in skin and determining the effect of antibiotic-loaded SiNPs on the rate of infection. The goal of this work is to investigate SiNPs loaded with clindamycin and tetracycline antibiotics as a treatment for *Staphylococcus epidermidis* infection in our 3D-bioprinted dermal model containing human fibroblasts. We demonstrated that antibiotic-releasing SiNPs reduced the bacterial load in these 3D-bioprinted models. Additionally, this dermal model represents a step forward in infection modeling for antimicrobial testing and offers a scalable and ethical testing platform compared to animal models.

## 2 Materials and methods

### 2.1 Synthesis of SiNPs

SiNPs were made by reacting 7.5 mL of cyclohexane, 1.8 mL of hexanol-1, and 1.7 g of Triton X-100 and stirring them at 800 rpm for 1.0 h. This solution was then mixed with 500 µL of dH_2_0 and 100 µL of ammonia >99%. The particles were allowed to sit for a period of 1 h while stirring, and tetraethyl orthosilicate (TEOS) > 98% was added at a volume of 100 µL to the solution. This mixture was allowed to stabilize, and then 100 µL of TEOS was added to increase the particle size. The particles were left spinning, covered in aluminum foil, for 3 days. The final volume added was ≈300 µL. The solution was opened, and the excess of ammonia was allowed to evaporate. The solution was centrifuged at 10,000 rpm for 10 min to remove unreacted reactants. The supernatant was removed from the pellet. The pellets were washed with 0.1 M HEPES buffer and centrifuged three more times at 10,000 rpm until the particles were removed from excess organic solvents. The pellets were resuspended in HEPES buffer and then stored in the fridge at 4 °C for a period of 8 h. SiNPs were always made fresh before experiments were conducted (Brito-Silva et al., 2013; Sobral-Filho et al., 2017). Transmission electron microscopy (TEM) was used to image the particles, and these images were acquired in a Jeol 1011 transmission electron microscope.

### 2.2 Antibiotic incubation with SiNPs

The SiNPs were removed from the 4 °C fridge and centrifuged at 8,000 rpm for 10 min. An aliquot of 1.0 mL of freshly prepared 0.1 M HEPES was added to the centrifuged tube, and the tubes were suspended on a floating foam raft in a sonication water bath for 30 min at 80 kHz in ice water at a temperature of ≈4 °C–8 °C. The tubes were then removed, and the particles were centrifuged at 5,000 rpm for 2 min with the supernatant discarded. A solution was made using two different kinds of antibiotics: clindamycin hydrochloride and tetracycline hydrochloride, both with purities greater than >99%. The antibiotic removed from −20 °C freezer was dissolved in 0.1 M HEPES buffer (1.14 g in 40 mL dH_2_O), and the antibiotic was kept on ice for the duration of the incubation with the SiNPs. The concentration of 200 mg/mL for both antibiotics was added at a 1:1 volume with the SiNPs to create a final concentration of 100 mg/mL for this specific concentration gradient. The SiNPs were kept at 4 °C on a rotary shaker at 300 rpm for 24 h after incubation with the antibiotic. The SiNPs were used immediately. Every experiment was conducted using freshly prepared particles produced in batches of >100 mL (Gounani et al., 2018). The particles were initially stored in PBS buffer, but this caused the particles to disintegrate. The SiNPs were put into microtubes and then centrifuged at 10,000 rpm for 5 min to spin down the particles, and then the supernatant was removed and discarded. Various concentrations of antibiotics were then tested as the optimal loading capacity was determined, ranging from 500 mg to 8 µg of clindamycin hydrochloride (HCl) and tetracycline HCl. The amounts of antibiotic were weighed on a top-loading scale, the material was transferred to a clean microtube, and 1.0 mL of HEPES buffer was added to the vial. Serial dilutions were performed if needed. The dose range was selected to mimic an oral dosage of the antibiotics.

#### 2.2.1 SiNP-tetracycline and SiNP-clindamycin release analysis

The SiNPs were then sonicated at 80 kHz for 20 min, followed by centrifugation at 10,000 rpm for 5 min. The supernatant was removed, and the particles were resuspended in fresh, sterile 0.1 M HEPES buffer that was filtered through 0.2-μm syringe filters. The supernatant was placed in microtubes that were previously autoclaved and then run on high-performance liquid chromatography (HPLC) to determine if the supernatant contained the desired antibiotic. Two standards were run on the HPLC, one each for tetracycline and clindamycin. A standard curve was run for clindamycin, as it was the antibiotic of choice. The samples were run in triplicate for each group: clindamycin SiNP-loaded, tetracycline SiNP-loaded, and SiNPs alone (no antibiotic in the supernatant samples).

The samples were run on the HP 1100 HPLC system that was constructed with a quaternary pump, a flow-through needle sample manager, a column selection module with an oven compartment, a diode array detector (DAD), and a fluorescence detector. All solvents were HPLC grade. The flow rate was set to 1.0 mL/min, and the injection was set to 20 μL. The column used was a Kinetex column (2.6 μ, C18, 100 Å) with dimensions of 4.6 mm × 150 mm. The column was kept at room temperature. The DAD was set to scan from 190 nm to 440 nm, and the system was set to collect a signal at 210 nm with a bandwidth of 10 nm. Solvent C was set as Optima™/Chromasolv acetonitrile (Fisher Chemical) while solvent D was set as Milli-Q™ water (Millipore) with 0.1% Optima™ trifluoroacetic acid (Fisher Chemical). The run started at 90% solvent D isocratic for 1 min, and then a gradient set to 50% of solvents C + D at 6 min, followed by another gradient to 90% of solvent C at 10 min, and then held isocratically for 1 extra min. The column was conditioned back to original conditions by reverting to 90% of solvent D using a gradient from 11 min to 13 min and then held for 5 min to completely flush the column for a total run time of 18 min. Once the HPLC was completed, the data were examined to determine if the peaks that were present were within a margin of error to confirm that the antibiotic was present after iterations of washing the particles. The HPLC with UV detection data were collected using a 90% aqueous mobile phase with 10% acetonitrile. This was followed by a gradient to 90% ACN with 10% water while the stationary phase was a C18 reversed-phase silica gel. The antibiotics were incubated with the SiNPs for a period of 24 h and then were washed 3× in HEPES buffer. The supernatant was then removed after the third wash and run on HPLC. The data were examined, and the following controls were run: bare SiNPs, clindamycin HCl standard, tetracycline HCl standard, and HEPES buffer. Samples were run in triplicate to ensure reproducibility.

### 2.3 Culturing *Staphylococcus aureus*

*Staphylococcus aureus* ATCC 27217 (ATCC, Virginia, US) and *Staphylococcus epidermidis* (obtained from the Biochemistry and Microbiology Department, University of Victoria) were cultured in Luria–Bertani (LB) broth (Thermo Fisher Scientific, Massachusetts, US). Bacteria were mixed with 2.0 mL of LB broth in a clean glass test tube and placed in a 37 °C biological incubator for an 18 h period to create an overnight broth culture. The bacteria were then expanded by taking 1.0 mL and adding it to 9.0 mL of fresh LB broth that had been previously autoclaved. The broth tube was placed in the incubator at 37 °C at 300 rpm for 2–4 h to obtain the exponential phase of bacterial growth of OD_600_ 0.2–0.4, determined after growth cultures were taken at different ratios. Aliquots (1.5 mL) were removed and placed in a spectrophotometer blanked with the sterile LB broth. The aliquot of bacteria was placed in the clean cuvette, and the OD_600_ value was given as a value between 0 and 2. The OD_600_ was determined in the exponential phase of growth of the bacteria by comparison to literature values of *Staphylococcus aureus* OD_600_ over a period of 24 h.

### 2.4 Bacterial minimum inhibitory concentration (MIC) test for *Staphylococcus aureus*

The bacteria were inoculated by transferring aliquots with serological pipettes into autoclaved media bottles (Thermo Fisher Scientific, Massachusetts, US). Clindamycin hydrochloride (Hello Bio^®^, New Jersey, US) and tetracycline hydrochloride (Sigma-Aldrich, Missouri, US) were the two antibiotics tested in this experiment. Distilled water was used to make up the LB and toxin superantigen (TSA) (Thermo Fisher Scientific, Massachusetts, US) media. Bacteria were grown at 37 °C with shaking at 300 rpm for 4 h, then read at an OD_600_ of ≈0.70 (0.71 was the actual recorded value). Clindamycin HCl was diluted to a concentration of 8.0 mg/mL in HEPES buffer 0.1 M and kept on ice by weighing 8 mg of −20 °C clindamycin antibiotic and adding 1.0 mL of HEPES buffer 0.1 M. A 1.0 mL aliquot of the broth culture was placed in each well of a 12-well plate, and 1.0 mL of the clindamycin sample was added to the first well. 1.0 mL was removed and mixed into the second well using a clean sterile filter tip for each well. The serial dilution was finished by discarding the remaining contents after the last well was completed. The well plate was wrapped in parafilm and incubated at 37 °C in the bacteria incubator. The 12-well plate was then incubated for 24 h to allow the culture to grow, and the visual determination of the MIC was considered based on the qualitative appearance of growth/no growth that was present in each well.

### 2.5 Bacterial incubation with SiNP-loaded antibiotics

*Staphylococcus aureus* was incubated as described above, and 1.0 mL of the culture was plated into each well of a 12-well plate. Different amounts of clindamycin HCl (500 mg, 250 mg, 100 mg, and 62.5 mg) were diluted in 1.0 mL of 0.1 M HEPES buffer. SiNPs were previously sonicated at 80 Hz in a water bath sonicator filled with ice, ≈4 °C, for 30 min. The SiNP particles were then removed and incubated with 1.0 mL of the antibiotic solution and placed in a rotary incubator shaker set at 4 °C and 300 rpm for 24 h. After incubation, 1.0 mL of the antibiotic solution was added to the culture broth and incubated in the bacterial incubator for 24 h at 37 °C, with shaking at 300 rpm. The plate was placed in the Cytation 5 plate reader, and the absorbance was read at OD_600_ to determine whether the bacterial concentration had decreased after the incubation with the antibiotics.

### 2.6 Culturing fibroblasts

Fibroblast cells (Primary Dermal Fibroblast Normal; Human, Neonatal (HDFn), ATCC^®^ PCS-201-010™—Neonatal foreskin fibroblasts, male donor) were used. Cells were transferred into a T75 flask (Thermo Fisher Scientific, Massachusetts, US) containing 15 mL of fibroblast growth media (DMEM, high glucose, no glutamine, no phenol red (Thermo Fisher Scientific, Massachusetts, US) + fetal Bovine Serum (FBS) certified 15% + 1% GlutaMAX (Thermo Fisher Scientific, Massachusetts, US), and Hanks Balanced Salt Solution (HBSS) (Thermo Fisher Scientific, Massachusetts, US) in a biological safety cabinet (BSC). The T75 flask containing cells was placed in a 37 °C, 5% CO_2_ incubator overnight. Cell cultures were monitored using phase-contrast microscopy. After initial cell culture, 1.0 mL of trypsin was added for cell passage. Cells were maintained by replacing the culture media every 3 days.

### 2.7 3D bioprinting of fibroblasts into constructs

**FIGURE 1:**
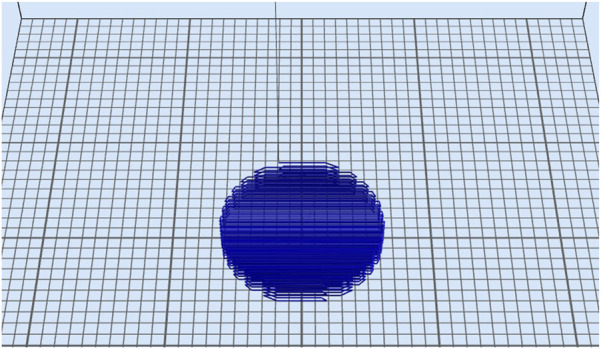
3D-bioprinted CAD file that was used to print the fibroblast construct. The final dome-shaped structure had a 1.0 cm diameter and six layers of fibers with an average width of ≈1.1 cm and a height of ≈0.7 cm (Aspect studio software, V1.2.59.0, Aspect Biosystems, Vancouver, BC, Canada).

The fibrin-based bioink was prepared based on previously published articles (Abelseth et al., 2019; Benwood et al., 2023; de la Vega et al., 2018; Sharma et al., 2020). The mechanical and rheological properties of this bioink were reported previously (Sharma et al., 2021). Fibroblasts were resuspended in a fibrin-based bioink at a concentration of 1.0 × 10^6^ cells/mL. Dome-shaped constructs were bioprinted based on the specifications detailed in the relevant CAD file (Figure 1) generated using Aspect’s studio software (V1.2.59.0, Aspect Biosystems, Vancouver, BC, Canada) using a rectilinear infill pattern in a repeated layer-by-layer fashion. The constructs consisted of six deposited layers of cell-laden bioink, according to previous studies (Sharma et al., 2020). Specific pressures are applied to each channel to monitor the flow rate to provide sufficient time for the crosslinking reaction to occur. The bioprinting speed was 25 mm/s, and the pressures for bioink, crosslinker, and buffer channels were 50 mbar, 60 mbar, and 100 mbar, respectively. The final structure comprised a 1.0-cm-diameter dome with six fiber layers, with an average width of ∼1.1 cm and a height of ∼0.7 cm.

The RX1 was used to 3D bioprint skin constructs containing fibroblasts in each well of a 12-well plate, and then 2.0 mL of fibroblast media was added to each well. Fibroblast media was made by adding 34 mL of phenol-free DMEM to a sterile conical and adding 15 mL of FBS and 1.0 mL of 1% GlutaMAX. Fresh media was prepared for each experiment. The constructs were stained with the CellMask™ Deep Red Actin Tracking Stain (Thermo Fisher Scientific, Massachusetts, US) according to the manufacturer’s instructions after printing. This process allowed for visualization under the confocal microscope using the appropriate excitation and emission.

### 2.8 Bacterial incubation with SiNP-loaded antibiotics and 3D-bioprinted fibroblasts

**FIGURE 2:**
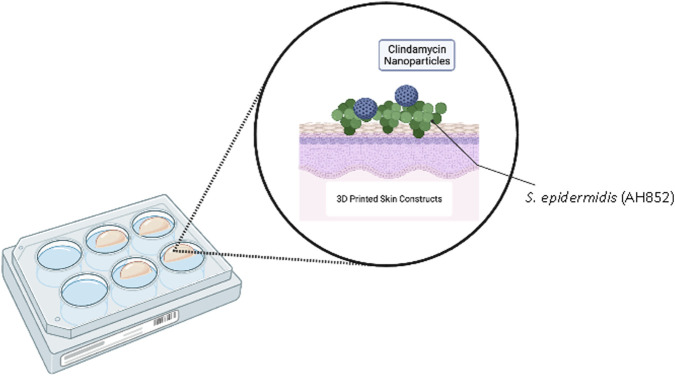
Schematic showing 3D bioprinting of human fibroblasts with the antibiotic clindamycin in SiNPs and *S. epidermidis*. The same setup was used to test tetracycline-loaded SiNPs and *S. aureus*.

*Staphylococcus aureus* was grown to an OD_600_ as described previously. SiNPs loaded with either clindamycin or tetracycline were spun down in a centrifuge at 10,000 rpm and washed three times with equal amounts of 0.1 M HEPES buffer (Figure 2).

The constructs were moved with a spatula to three separate plates: one for clindamycin, one for tetracycline, and one for no treatment. The 12-well plate was opened, and 1.0 mL of the overnight broth culture of *S. aureus* at OD_600_ ≈ 0.70 was added to each well and placed at the side of the culture to allow the bacteria to disperse over the construct. The construct was moved to the Cytation 5 plate reader for incubation of the bacteria/fibroblast mixture with 5% CO_2_ and was left for a period of 4 h at 37 °C. After the incubation time, the 12-well plate was taken into the BSC, 1.0 mL of each antibiotic-loaded-SiNP in 0.1 M HEPES buffer was added to the wells, and the plate was placed back in the incubator for 6 h. The BacLight™ LIVE/DEAD^®^ Bacterial Viability Kit Protocol (kit L13152, Thermo Fisher Scientific, Massachusetts, US) was freshly prepared according to the manufacturer’s instructions. A 1.0 mL aliquot of the final solution was added to each well. Fluorescence levels were determined using the Cytation 5 plate reader using the Texas Red filter and the GFP filter after 15 min of incubation wrapped in aluminum foil. The plate was then fixed with 1:1 methanol-free 4% formaldehyde in pre-sterile PBS and filtered. The well plate was then read on the confocal microscope by analyzing the excitation at 485 nm and 530 nm for the emission of the green fluorescence from Syto9, and 485 nm excitation and 630 nm emission of the red fluorescence from the propidium iodide. Zen Black software was used to image the bacterial constructs, and Zen Blue software was used to process the images and interpret the data.

### 2.9 Statistical analysis

One-way ANOVA (analysis of variance) and Tukey’s post-hoc test were performed for colony-forming units (CFUs) counting using GraphPad Prism 6.0 (GraphPad Software, Inc., La Jolla, CA, USA), which was also used to depict all graphs. A p-value <0.05 was considered statistically significant. Data are presented as the mean ± standard error of the mean.

## 3 Results and discussion

### 3.1 SiNPs loaded with antibiotics

**FIGURE 3:**
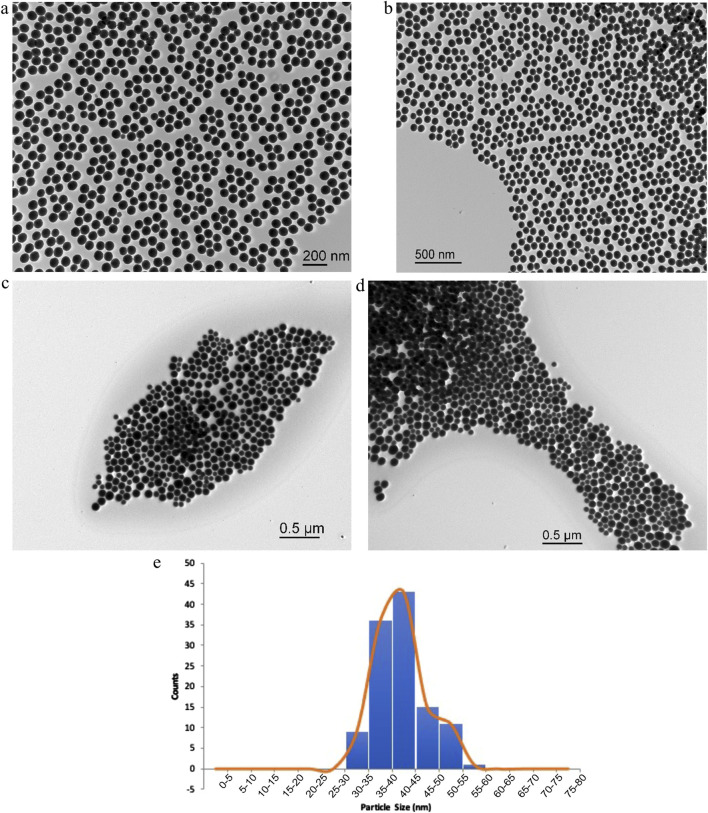
**(a,b)** SiNPs were prepared and imaged on TEM in 10 mL of ethanol. **(c,d)** SiNPs prepared by synthesis with increased ammonia. **(e)** Histogram of SiNPs in **(c,d)** after synthesis and 3× HEPES washing solution placed in ethanol and imaged on TEM. Number of counts n = 115 to determine the size distribution of SiNPs after preparation.

SiNPs were synthesized and imaged using TEM by diluting them in ethanol and placing them on a TEM grid (Figures 3a,b). The images were then analyzed on ImageJ software to determine the distribution size histogram, as shown in Figure 3e.

The size distribution of the particles mainly fell in the 40–50 nm target after preparation (Figure 3e). The size was controlled by the injection of TEOS, which increased the amount of orthosilicic acid in the system. Increasing size leads to decreased toxicity, as detailed by Mannerström et al. (2016). Figures 3c,d show the greater polydispersity of the particles.

The buffer of choice for the nanoparticles needed to have no ultraviolet light absorbance, as HPLC was later used to detect the antibiotics of interest, which have a peak at ≈220 nm. There has been some analysis of the cytotoxic effects of light-exposed HEPES buffer due to the formation of hydrogen peroxide (Devlin et al., 2021; Liu et al., 2022; Rahaman et al., 2023). This limitation was overcome during the experimentation by creating a fresh solution before the experimentation. The solution was kept in a conical flask wrapped in aluminum foil to reduce the light able to penetrate the buffer. The buffer has membrane impermeability that is necessary because it will not penetrate the cells or the bacterial membrane (Hu et al., 2017). The particles were washed 3× in an aqueous solution of pH 7.0 adjusted with a HEPES buffer balanced with sodium hydroxide.

### 3.2 Phenotypic characterization of *S. aureus* and *S. epidermidis*

**FIGURE 4:**
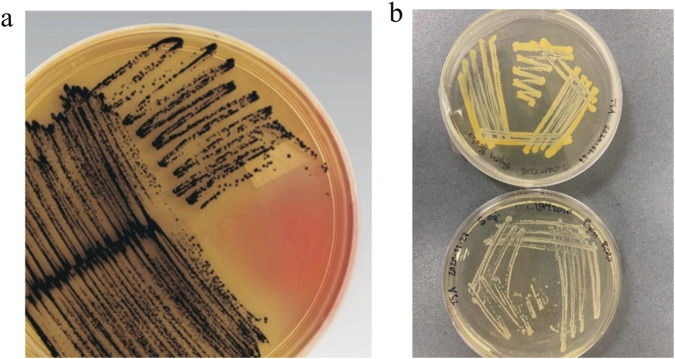
**(a)** Vogel and Johnson agar (VJA) culture of *S. aureus* (Thermo Fisher Scientific). **(b)** Top plate: *S. aureus*; and bottom plate: *S. epidermidis*, both grown on TSA at 37 °C for 18 h.

The bacterial cultures were streaked onto Vogel and Johnson agar (VJA), which is classically used in diagnostics using plating methods rather than genomics to ensure species typing. The presence of black colonies after incubation for a maximum of 24 h at 37 °C is a positive indication of *S. aureus*, along with a yellow border. Figure 4a illustrates the plating technique that showed the red agar turning black/yellow. *S. epidermidis* was also cultured in VJA, and the presence of pink media was seen behind the black colonies, which can qualitatively distinguish the species.

*Staphylococcus aureus* can be diagnosed in several ways, depending on the type of infection and location. The initial step involved is the Gram stain test, which will indicate a Gram-positive species of bacteria that will appear in clustered cocci form (Prasad et al., 2012). The next plating technique was done on MacConkey agar to examine the lactose-fermenting abilities of bacteria. It is categorized as a selective and differential medium (Sigma-Aldrich). The agar was used in this study to examine the difference between *Staphylococcus aureus* and *Staphylococcus epidermidis*, the two bacterial species used. *Staphylococcus aureus* can ferment lactose, causing the pink plate to turn yellow. In contrast, *Staphylococcus epidermidis* cannot ferment lactose, and the plate will stay pink. This test was completed for both species grown on the same plate at 37 °C for 24 h to confirm the identity of both species.

The VJA agar and MacConkey agar were subcultured from the original TSA plates. Other testing, such as the coagulase test, was done on both species, and *S. aureus* was found to be positive, while *S. epidermidis* was negative. Figure 4b shows that *S. aureus* grown on TSA had a characteristic yellow color when streaked (top), and *S. epidermidis* had white colonies (bottom). This was further accompanied by a literature study that illustrated the name “aureus,” meaning gold, characterized the 2–3 mm diameter circle of gold pigment produced by the colonies of *S. aureus* (Pal et al., 2023). This contrasted with the small white colonies 1–2 mm in diameter. This identification was found to be consistent with all TSA plating. This analysis confirmed the species of bacteria as the species of interest and, for the purposes of this study, that they could be compared in the analysis. Antibiotic sensitivity testing was conducted on the primary species of interest, *S. aureus*, grown in a confluent layer with two antibiotic discs, tetracycline and vancomycin, respectively. The disks were placed on TSA agar plates and then positioned in a bacterial incubator for 24 h (Supplementary Material S1) to confirm susceptibility.

### 3.3 Characterization of antibiotic-laden nanoparticles

This study selected two antibiotics of interest due to their molecular structure and current treatment of *S. aureus* bacterial infections. The samples were run on HPLC to determine if the antibiotic was observed after the washing stages. Antibiotic standards were run in buffer to determine the peaks of interest for clindamycin HCl and tetracycline HCl. The HEPES buffer was also run. Samples were run in triplicate (clindamycin-loaded SiNP supernatant, tetracycline-loaded SiNP supernatant, and bare SiNPs not loaded with antibiotics) (Supplementary Figure S2). The HPLC detection of clindamycin post incubation and washing in the supernatant of SiNP data is presented in Table 1.

**TABLE 1:** HPLC detection of clindamycin post-incubation and washing of the SiNP supernatant.

Sample retention time (min)	Retention time (min)
Clindamycin standard in HEPES buffer 200 mg/mL	6.286
Clindamycin 500 mg/mL supernatant in HEPES buffer after incubation with SiNPs	6.209
Clindamycin 200 mg/mL supernatant after incubation with SiNPs	6.191
HEPES buffer	0

The presence of the antibiotic could be observed in the data sets obtained by HPLC analysis. As a control, an initial weight sample of clindamycin HCl in 500 mg/mL in water was used to determine whether any peaks were present and to determine the mobile phase. Clindamycin HCl was also tested in ethanol and in buffer. Due to the polarity and hydrophobicity of the molecule, it was difficult to determine the optimal pH range, which was achieved by adding 1–2 sodium hydroxide pellets into the buffer to neutralize the solution to ≈ pH 7.5, which was confirmed using pH strips. Further analysis was completed to create a standard curve using five standards. This was challenging, as saturation of the antibiotic in the buffer solution was difficult to overcome. Clindamycin was determined to be optimal in the HEPES buffer at 14.2 mg/mL, and this was then serially diluted by adding 500 μL of this original standard to 500 μL of fresh buffer that was prepared on the day of the experiment.

The presence of clindamycin HCl was verified using the HPLC method due to the lack of specificity and accuracy of UV-Vis spectrophotometry (Devlin et al., 2021; Liu et al., 2022; Rahaman et al., 2023). The amount of antibiotic was quantified using a calibration curve to detect the presence of clindamycin HCl recovered in a sample. This method of recovery extraction is needed to determine the amount of loss of antibiotic due to washing and to determine the loading capacity and saturation levels. The loading of clindamycin HCl into the SiNPs was consistent across all batches, as confirmed by reproducible concentration profiles obtained in triplicate measurements over multiple iterations. The antibiotic peak was clearly identified within the expected range, confirming successful loading and detection. Quantitative HPLC analysis verified that the concentration of clindamycin HCl in the particles was stable and measurable within the linear range of the calibration curve. The indication that the particles were exhibiting the same levels of antibiotic as 200 mg/mL in buffer suggests that the particles had reached their loading capacity. While this method indicates that antibiotics were successfully loaded into the SiNPs, it does not provide insight into the time course of release.

### 3.4 3D bioprinting of human dermis and its infection with bacteria

**FIGURE 5:**
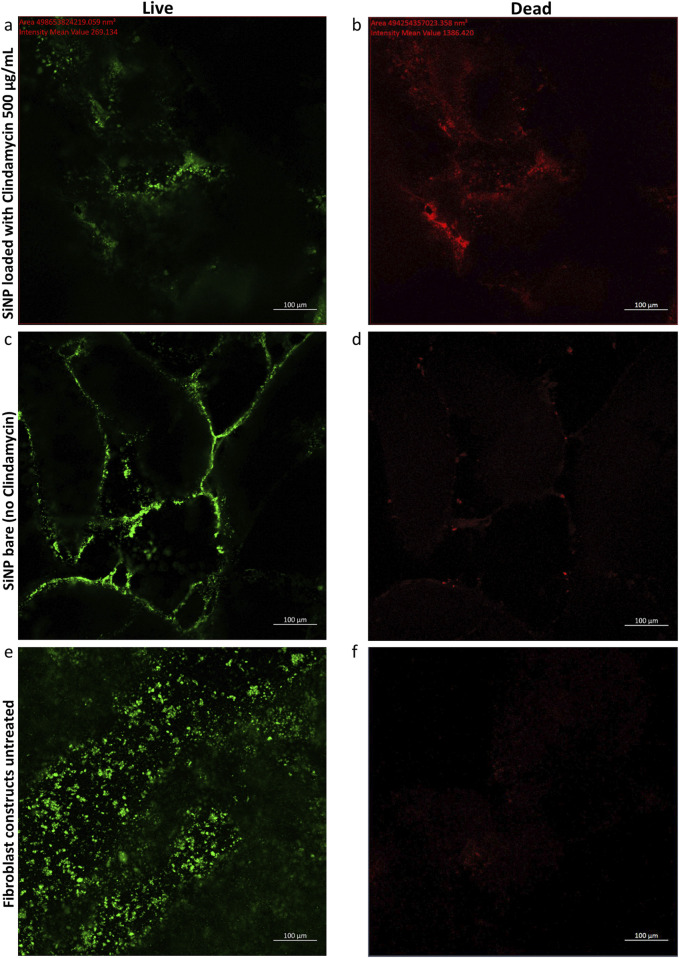
*S. aureus* bacterial fluorescence imaging using BacLight^®^. Bacteria were imaged in LB broth after the addition of SiNP-loaded clindamycin 500 mg/mL: **(a)** green emission (live bacteria) and **(b)** red emission (dead bacteria). Fibroblast treatment of bare SiNPs: **(c)** green emission (live bacteria) and **(d)** red emission (dead bacteria). Untreated fibroblast constructs: **(e)** green emission (live bacteria) and **(f)** red emission (dead bacteria). Images **(a–f)** are from the same location with different fluorescence.

**FIGURE 6:**
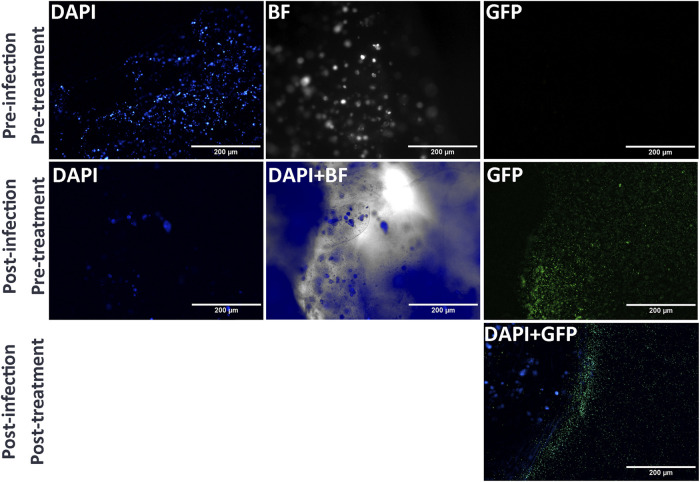
Imaging of 3D-bioprinted construct of fibroblasts infected with *S. epidermidis (AH852)* and treated with SiNP-loaded clindamycin 500 mg/mL under three conditions: pre-infection and pre-treatment (control group, with only fibroblasts 3D bioprinted), post-infection and pre-treatment (only *S. epidermidis* with GFP (green dots in the image) inoculation in the 3D-bioprinted construct, but with no treatment), and post-infection and post-treatment (*S. epidermidis* with GFP (green dots in the image) inoculation in the 3D-bioprinted construct treated with SiNP-loaded clindamycin 500 mg/mL).

The primary function of fibroblasts is to maintain the structural integrity of connective tissues by supporting the extracellular matrix. This cell line was chosen because it mimics the natural infection site of the *S. aureus*. 3D-bioprinted fibroblast constructs were used to illustrate an *in vitro* analysis to show Staphylococcal infection on the constructs. Once infected, the constructs can show the effectiveness of the treatment in patients with serious skin infections (Dokoshi et al., 2025; Kendall and Feghali-Bostwick, 2014). Figure 5 shows the green fluorescence signal, indicating live bacteria (Figures 5a,c,e), and the red fluorescence signal, indicating dead bacteria (Figures 5b,d,f). The comparison demonstrates the ability of the SiNPs loaded with clindamycin 500 μg/mL to induce bacterial death. Figures 5c,d show that the bare SiNPs did not have an impact on bacteria death, indicating the particles were non-toxic drug carriers. Figure 6 show *S. aureus* bacterium incubated without the use of antibiotics or particles, which gave a green fluorescence signal, indicating viability. The use of this control was significant because the fluorescence kit that was used is specific for bacteria and illustrated that the bioprinted construct did not have any natural antimicrobial properties, as was anticipated.

The constructs inoculated with bacterial cultures were labeled with green fluorescent protein (GFP) and then were treated with antibiotic-loaded SiNPs. The bacteria were transformed to express GFP, as done previously, to enable easy visualization (Devlin et al., 2021; Liu et al., 2022; Rahaman et al., 2023; Yang et al., 2022). GFP labeling enabled us to track the presence of the bacteria. Different conditions were investigated for the bioprinted constructs, including pre-infection and pre-treatment. The pre-infection group corresponded to a control group of bioprinted fibroblast constructs with no bacteria. The post-infection and pre-treatment group corresponded to the bacterial inoculation in the 3D-bioprinted construct prior to treatment with SiNPs, and the post-infection and post-treatment group corresponded to the bacterial inoculation in the 3D-bioprinted construct with SiNP-loaded clindamycin at a treatment of 500 mg/mL. As expected, the third group had fewer bacteria, as indicated by GFP expression, due to the effective action of the SiNP-loaded clindamycin treatment in the “dermis” model of 3D-bioprinted construct against *S. epidermidis* (Figure 6).

*Staphylococcus epidermidis* was used as a model organism for a live, real-time analysis that would allow for experimentation of the *in vitro* modeling to determine if saturation times >24 h would improve the SiNP release, in contrast to the 4–6 h that was completed in this study.

**FIGURE 7:**
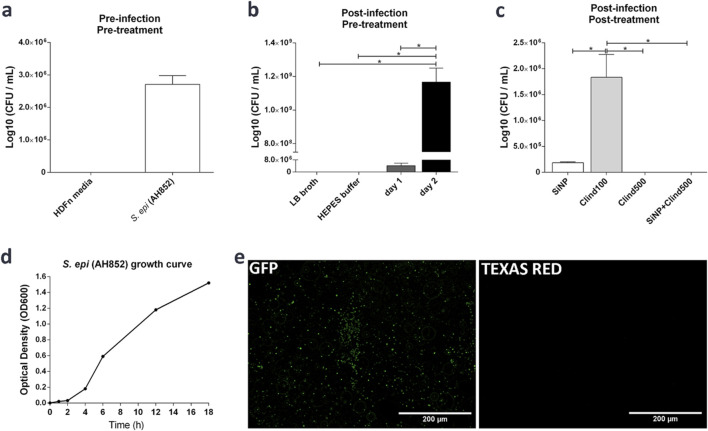
Investigations in the “dermis” model of 3D-bioprinted construct inoculated with *S. epidermidis* and treated with SiNP-loaded clindamycin 500 mg/mL. **(a)** CFU counts in the pre-infection and pre-treatment condition, showing the higher *S. epidermidis* CFUs (before inoculating them in the 3D-bioprinted construct) than in the fibroblast HDFn media. **(b)** CFU counts in the post-infection and pre-treatment conditions show higher *S. epidermidis* growth over time. **(c)** CFU counts in the post-infection and post-treatment conditions highlight the effectiveness of SiNP-loaded clindamycin 500 mg/mL treatment against *S. epidermidis* in the 3D-bioprinted construct. **(d)** Growth curve of the *S. epidermidis* (AH852) showing its growth over time. **(e)** *S. epidermidis* bacterial imaging using BacLight® fluorescence detection. Bacteria were imaged in LB broth after the addition of SiNP-loaded clindamycin 500 mg/mL, green fluorescent protein (GFP): live bacteria (green dots in the image), Texas Red: dead bacteria (red dots in the image). The absence of red dots indicates that the SiNP-loaded clindamycin 500 mg/mL was an effective treatment against *S. epidermidis* in the 3D-bioprinted construct, in that it prevented the *S. epidermidis* from forming colonies or biofilms in the 3D-bioprinted construct. Images on **(e)** are the same spot with different fluorescence (one-way ANOVA and Tukey post-test; *: p < 0.05).

Experiments were also performed to quantify the effectiveness of the previous experiment in the “dermis” model of 3D-bioprinted construct inoculated with *S. epidermidis* and treated with SiNP-loaded clindamycin 500 mg/mL, mimicking the real infectious skin disease. Figure 7a showed that the CFU counts in the pre-infection and pre-treatment conditions had higher *S. epidermidis* CFUs than in the HDFn media. In this test, the results were regarding the timeframe before the bacteria were inoculated in the 3D-bioprinted construct, as the bacteria were separated from the fibroblast media, which confirms a lack of contamination in the 3D-bioprinted construct that could change the results. Figure 7b shows the CFU counts in the post-infection and pre-treatment condition, indicating higher *S. epidermidis* growth on day 2 post-infection. The growth was uncontrolled, as it was untreated. Figure 7c shows the CFU counts in the post-infection and post-treatment conditions, highlighting the effectiveness of SiNP-loaded clindamycin 500 mg/mL in killing *S. epidermidis* in the 3D-bioprinted construct. It was similar to the treatment with clindamycin at 500 mg/mL with no SiNP delivery, which was also favorable. However, the treatment with clindamycin at 100 mg/mL was not effective, showing considerable CFUs. Also, SiNPs alone showed some *S. epidermidis* growth. Figure 7d shows the growth curve of the *S. epidermidis* over time. Figure 7e shows bacterial imaging of *S. epidermidis* using BacLight^®^ fluorescence detection. Bacteria were imaged in LB broth after the addition of SiNP-loaded clindamycin 500 mg/mL. The GFP-stained live bacteria are the green dots in the image, and the Texas Red-stained dead bacteria are the red dots in the image. The absence of red dots indicates that the SiNP-loaded clindamycin 500 mg/mL prevented the *S. epidermidis* from forming colonies or biofilms in the 3D-bioprinted construct.

## 4 Conclusion

Here, we demonstrate how using SiNPs to locally distribute antibiotics can treat bacterial infection effectively in a 3D-bioprinted skin model. Significant cell death occurred in the samples incubated with SiNP-loaded clindamycin HCl. The use of unloaded SiNPs illustrates that there was little to no effect on the bacteria without the addition of the antibiotic. This is important, as other types of nanoparticles that are used for drug delivery, such as silver or gold, can have an antimicrobial effect on their own. Silver can be very beneficial against certain bacteria. However, there can be some toxicities associated with the introduction of silver colloids into the body. *S. aureus* was able to grow without the introduction of antibiotics, and when untreated, did not give any red signal with propidium iodide. Some cell death occurred in the experiment with clindamycin HCl present in solution. However, this effect was comparable to SiNPs loaded with the same initial loading concentration of 500 μg/mL. The loss of the antibiotic in the washing step must be factored for the samples treated with SiNPs. This effect suggests there was a better impact with the use of SiNPs, as there is longer contact time. *Staphylococcus epidermidis* exhibited cell death after treatment with tetracycline HCl-loaded SiNPs compared to the bare SiNPs under the same conditions. The SiNPs were loaded and optimized from two separate batches and then tested on HPLC prior to incubation. The use of a CO_2_ incubator at 5% was also important as it kept the constructs in a stable environment that would mimic the cell’s natural state. The temperature was kept at 37 °C, and the pH was between 7.0 and 7.5. This pH, however, was lowered after the incubation of bacteria, creating a more acidic environment that was tested using pH strips and was determined to be between 4.5 and 5.5 after the conclusion of the incubation.

SiNPs impacted the ability of *Staphylococcus aureus* to proliferate. The bare SiNPs did not have a negative impact on the bacteria and did not display any inert antimicrobial activity against the bacteria. The use of solution-based antibiotics was slightly less effective in the qualitative analysis of the cell death of the bacteria. The use of 3D-bioprinted human dermis enabled biofilms to be visually identified. This model was deemed successful at representing the deep dermis layer when examining bacterial infection strategies. Our results demonstrated the successful application of nanomedicine as a promising strategy to combat antibiotic resistance. Furthermore, we established a 3D-bioprinted human dermis infection model mimicking skin infection to investigate the delivery of clindamycin and tetracycline using SiNPs, offering an ethical and cost-effective alternative to animal experiments, which often fail to accurately replicate human biology. These insights contribute to optimizing bioprinting approaches for infected skin tissue engineering and advancing nanomedicine strategies, highlighting their crucial role within a microfluidic 3D infected skin model. The use of *Staphylococcus epidermidis* as a model organism can enable a real-time analysis that would allow for experimentation of *in vitro* modeling to determine whether saturation times >24 h would improve the nanoparticles’ antibiotic release, in contrast to the 4–6 h that was used in this study. Overall, SiNPs show significant promise for delivering antibiotics for treating bacterial infections in the skin.

In our study, we investigated a skin-equivalent platform that combines antibiotic-loaded SiNPs as a treatment of a dermal bacterial infection in a 3D-bioprinted model, enabling physiologically meaningful evaluation of localized antimicrobial therapy. This system provides a more realistic model of investigation compared to 2D cultures and simple *in vitro* assays, bridging the gap between nanomedicine testing and human-like tissue models. We demonstrated functional antibiotic release within a 3D human tissue context, which is more relevant than testing such approaches in a monolayer culture. Our model can represent a step forward in infection modeling for antimicrobial testing while also offering a scalable and ethical testing platform. SiNP-based delivery improves drug stability and enables localized, sustained release at the site of infection compared with direct administration of free antibiotics. This approach enhances antibiotic retention within the construct, reduces burst release and off-target exposure, and may achieve effective antibacterial activity at lower doses. Such controlled delivery is particularly advantageous for localized infection models, such as 3D-bioprinted skin constructs (Slowing et al., 2007; Vallet-Regi et al., 2017).

## Data Availability

The original contributions presented in the study are included in the article/Supplementary Material; further inquiries can be directed to the corresponding author.
