# Supplementary material for: Using silica nanoparticles to deliver antibiotics for treating Gram-positive bacterial infections in a 3D-bioprinted dermal model
Source: Front Bioeng Biotechnol. 2026 Feb 17;14:1737616. doi: 10.3389/fbioe.2026.1737616 (PMC12953382; doi:10.3389/fbioe.2026.1737616)
Supplement: Supplementary file 1 [file Supplementaryfile1.docx]

**Supplementary Material**

Antibiotic sensitivity testing was conducted on the primary species of interest *S. aureus* that was grown in a confluent layer with two antibiotic disk, Tetracycline and Vancomycin respectively. The disks were placed on TSA agar plates and then positioned in a bacterial incubator for 24 h (Supplementary Material S1). Figure S1 illustrates the antibiotic disk diffusion and the zone of inhibition was measured using a ruler. This form of testing is called the Kirby-Bauer disk diffusion test and is based on the determination of sensitive or resistance pathogens based on the absence of growth around the antibiotic disk (NCCLS, 1995). The use of Mueller Hinton agar plates is the best candidates for the diffusion test based on composition allow efficient diffusion. However, TSA was deemed an acceptable alternate based on Brenner et al. (1972) that illustrated the appropriate uses of different agars for antimicrobial susceptibility testing.

The determination from the NCCLS provided the Performance standards for antimicrobial susceptibility testing that allowed for the interpretation of the results. The testing was conducted in duplicate to ensure accuracy of the results. The interpretation of the *S. aureus* pathogen incubated with tetracycline was measured at 19 mm. The classification was given to *S. aureus* as sensitive to tetracycline, due to the duplicate plate replication of the experimentation this was an accurate reporting as both plates reported the same value. However, this *S. aureus* contained a gene variant that had Tetracycline resistance to 25 μg. The analysis of the Vancomycin disk was determined to be 15 mm that indicates sensitivity to the antibiotic. It should be noted that the reliance of the Vancomycin test has been deemed to be variable based on this test alone and genomic analysis is recommended for the identification of resistant isolates to Vancomycin in *S. aureus* groups.

a

b


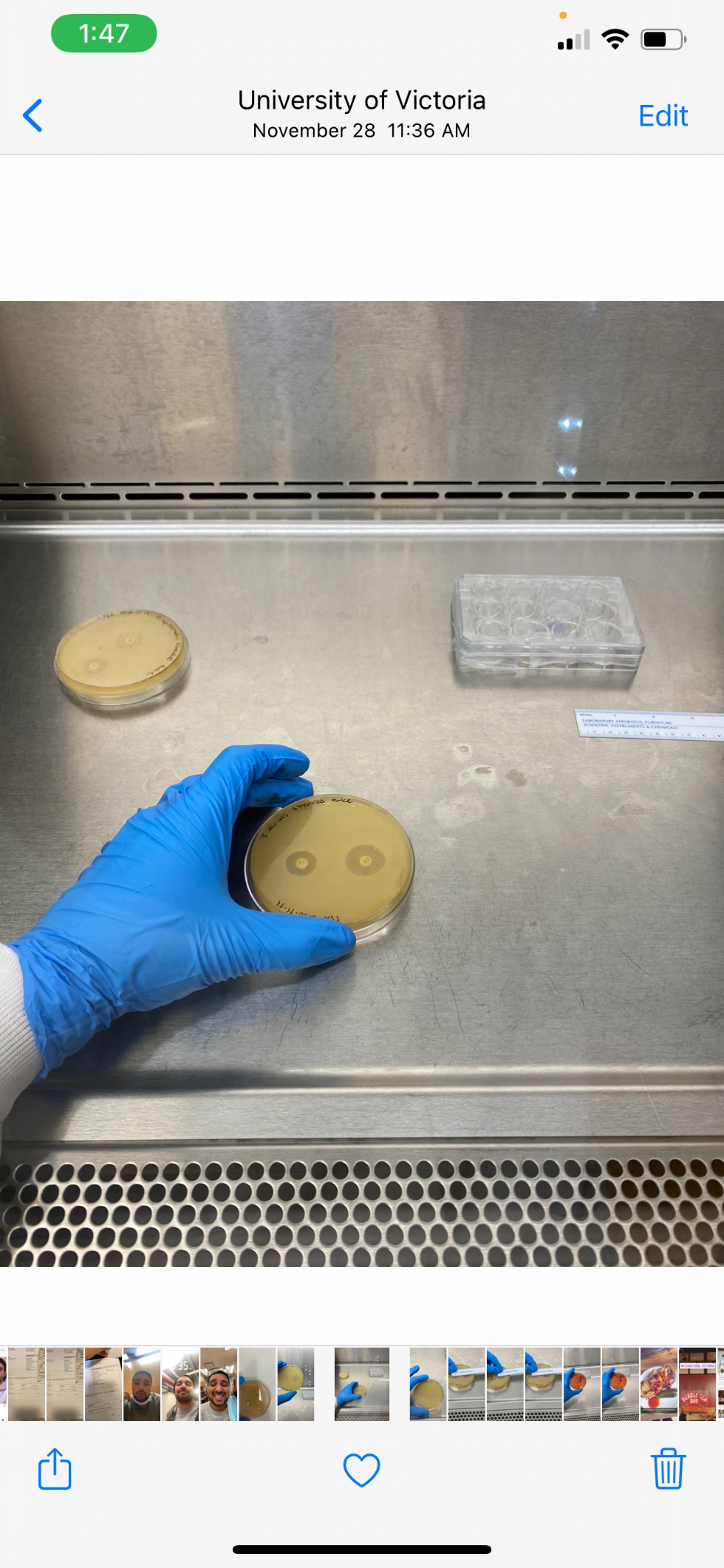

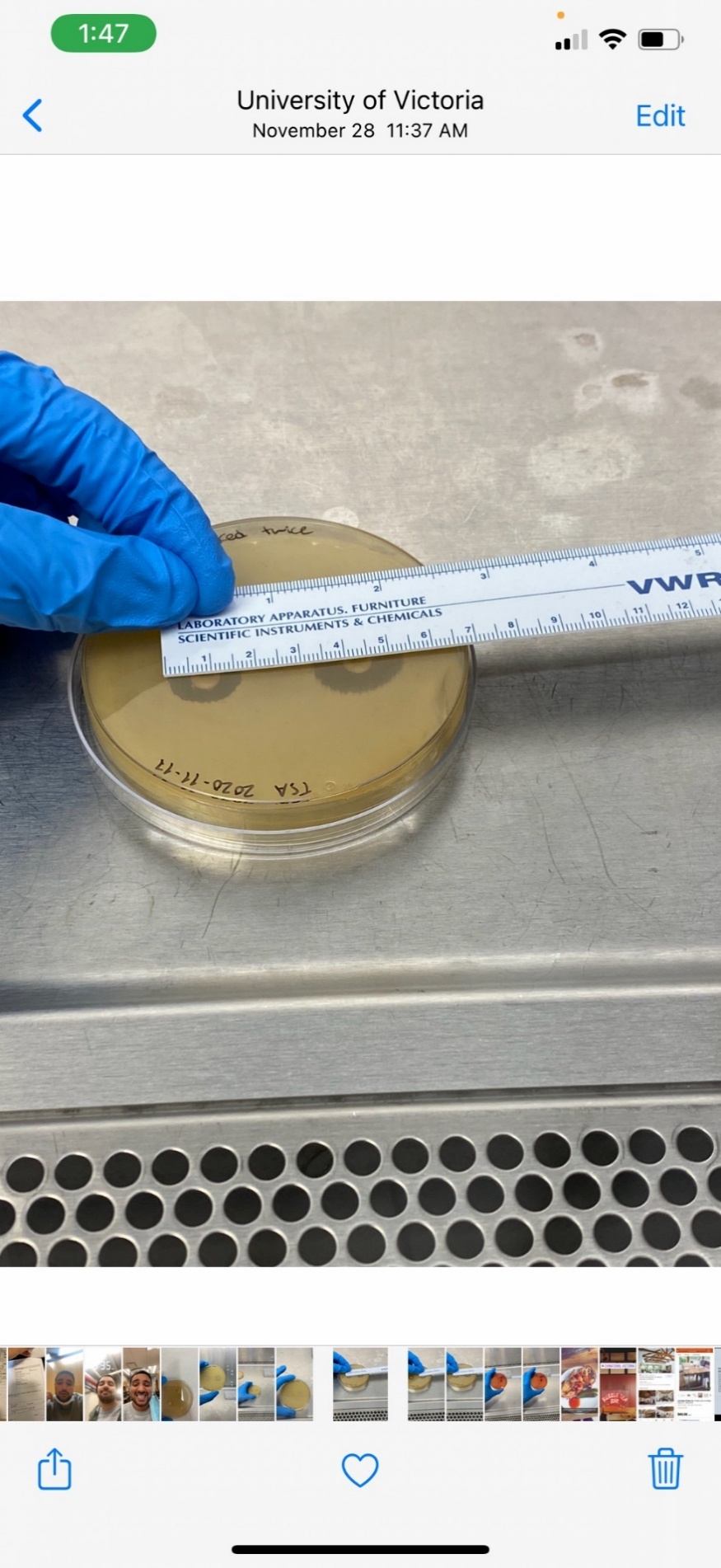


20 cm

20 cm

**Supplemental Figure 1.** *S. aureus* grown confluently on TSA agar with the addition of two antibiotic disks, Vancomycin (30 μg) and Tetracycline (30 μg). The measurement was done of the zone of inhibition and determined to be 15 mm for Vancomycin and 19 mm for Tetracycline.

**Supplemental Figure 2**. (a) 41 mg/mL HPLC of Tetracycline HCl antibiotic several peaks ranging from 5.222-7.641. (b) 7 mg/mL HPLC of Clindamycin HCl antibiotic peak observed at 6.286. (c) HPLC of SiNP not loaded with antibiotics (bare silica), no peaks observed in regions of interest for antibiotics. (d) HPLC of HEPES buffer no peaks were noted. (e) HPLC of 51 mg/mL of Tetracycline HCl loaded antibiotics loaded SiNP after 3x washing in HEPES buffer. (f) HPLC of 48 mg/mL Clindamycin HCl antibiotic loaded SiNP after 3x washing in HEPES buffer.


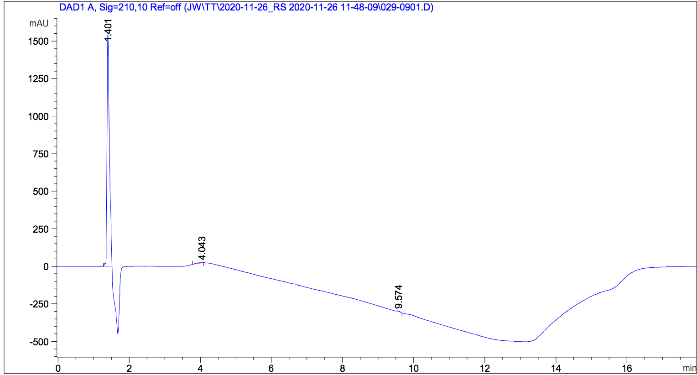

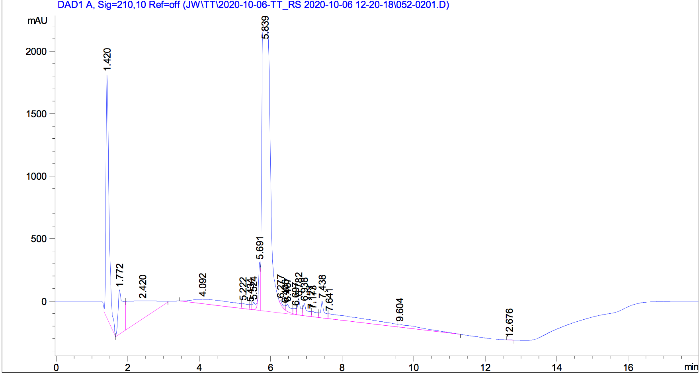

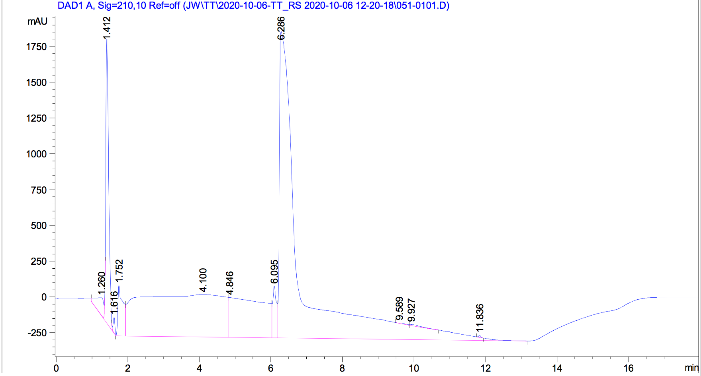


a

b


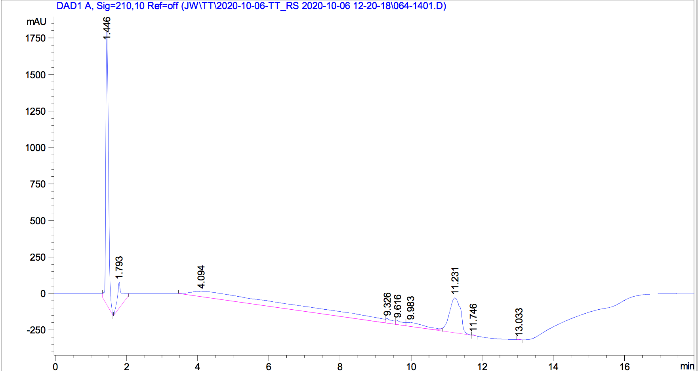


c

d


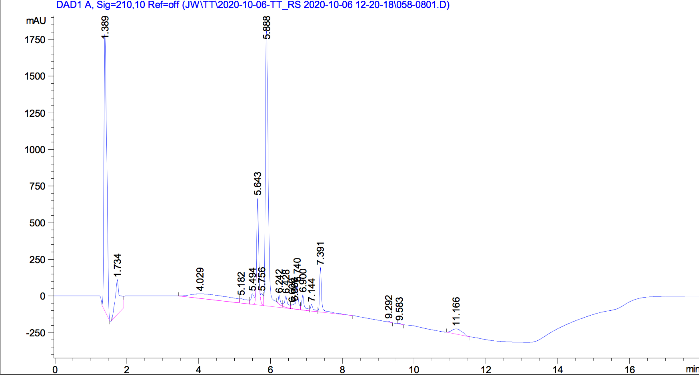


e


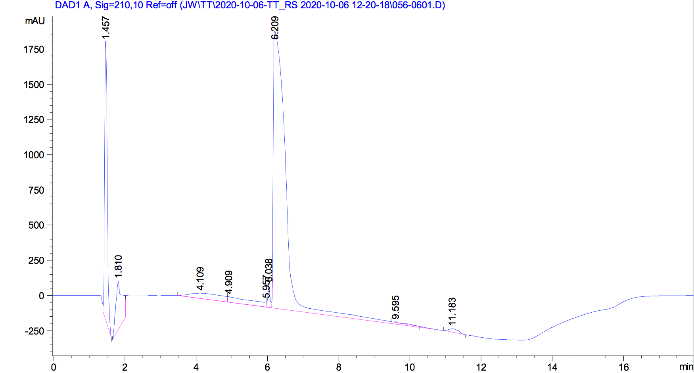


f

**41 mg/mL of Tetracycline**

**7 mg/mL of Clindamycin**

**HEPES buffer**

**SiNP bare (no antibiotic)**

**51 mg/mL of Tetracycline and SiNP**

**48 mg/mL of Clindamycin and SiNP**

**References**NCCLS. Performance standards for antimicrobial susceptibility testing, Sixth informational supplement. NCCLS document M100-S6, Villanova, Pa. 1995

Brenner VC, Sherris JC. Influence of Different Media and Bloods on the Results of Diffusion Antibiotic Susceptibility Tests. Antimicrobial Agents and Chemotherapy. 1972;1:116-122.
